# Stable Hydrogen Production from Ethanol through Steam Reforming Reaction over Nickel-Containing Smectite-Derived Catalyst

**DOI:** 10.3390/ijms16010350

**Published:** 2014-12-25

**Authors:** Hiroshi Yoshida, Ryohei Yamaoka, Masahiko Arai

**Affiliations:** Division of Chemical Process Engineering, Faculty of Engineering, Hokkaido University, Sapporo 060-8628, Japan; E-Mails: yoshida@chem.kumamoto-u.ac.jp (H.Y.); yamaoka.ryouhei@kao.co.jp (R.Y.)

**Keywords:** hydrogen, steam reforming, ethanol, nickel, smectite

## Abstract

Hydrogen production through steam reforming of ethanol was investigated with conventional supported nickel catalysts and a Ni-containing smectite-derived catalyst. The former is initially active, but significant catalyst deactivation occurs during the reaction due to carbon deposition. Side reactions of the decomposition of CO and CH_4_ are the main reason for the catalyst deactivation, and these reactions can relatively be suppressed by the use of the Ni-containing smectite. The Ni-containing smectite-derived catalyst contains, after H_2_ reduction, stable and active Ni nanocrystallites, and as a result, it shows a stable and high catalytic performance for the steam reforming of ethanol, producing H_2_.

## 1. Introduction

Fuel cells have been given attention as a clean technology for energy, and hydrogen is one of the most promising fuels due to many advantages, such as high energy efficiency, no emission of air pollutants and greenhouse gases and the presence of abundant H_2_-containing resources [1,2]. However, at present, hydrogen is mainly produced from fossil fuels by steam reforming of natural gas and light oil, and so, new and sustainable ways for hydrogen production are greatly required. Hydrogen production from biomass is an ideal means, and many researchers have investigated the steam reforming of ethanol, which can be produced by the fermentation of biomass [3,4,5,6,7,8,9,10,11,12,13]. Only CO_2_ and H_2_ are theoretically produced through steam reforming of ethanol (Equation (1)), but in practice, some other reactions can also occur and several intermediates and by-products formed. The steam reforming of ethanol (Equation (1)) mainly proceeds through several consecutive reactions (Equations (2)–(5)) [6]. A number of metal-supported catalysts have been investigated using nickel [3,4,5,6], cobalt [8,9,10] and noble metals [11,12,13,14], and low-cost nickel-based catalyst showed the highest catalytic performance among the transition metals examined so far.

(1) $$C_{2}H_{5}OH+3H_{2}O↔2CO_{2}+6H_{2}$$

(2) $$C_{2}H_{5}OH↔CH_{3}CHO+H_{2}$$

(3) $$CH_{3}CHO↔CH_{4}+CO$$

(4) $$CH_{4}+2H_{2}O↔CO_{2}+4H_{2}$$

(5) $$CO+H_{2}O↔CO_{2}+H_{2}$$

When Ni-based catalyst is used for the steam reforming of ethanol, the main problem is a catalyst deactivation by carbon deposition formed from several side reactions. Ethylene is formed by the dehydration of ethanol (Equation (6)); it is rapidly reduced to carbon species, which deposit on the catalyst (Equation (7)). The decomposition of methane (Equation (8)) and the Boudouard reaction (Equation (9)) also occur and cause the catalyst activity of Ni to decrease; hence, the suppression of these side reactions and carbon deposition is one of the most significant problems for the development of practical Ni-based catalysts for the steam reforming of ethanol.

(6) $$C_{2}H_{5}OH↔C_{2}H_{4}+H_{2}O$$

(7) $$C_{2}H_{4}\to 2C+2H_{2}$$

(8) $$CH_{4}\to C+2H_{2}$$

(9) $$2CO\to C+CO_{2}$$

Christensen *et al.* [15] used hydrotalcite as a support of Ni catalyst for the steam reforming of methane and investigated the influence of the crystallite size of Ni. They reported that a smaller Ni crystallite has higher resistance to coke formation compared to conventional Ni-supported catalysts. Muroyama *et al.* [6] prepared a Ni-containing material of NiAl_2_O_4_ and showed its high catalytic performance and stability. These studies suggest that the modification of the structure around Ni species is a key to the development of durable Ni catalysts with less carbon deposition. In the present work, a Ni-containing mesoporous smectite material has been synthesized and used as a precursor of Ni catalyst, which has been prepared through a certain reduction. For a comparison, another smectite material containing Mg was prepared and used as a support for Ni, which was loaded by impregnation. A few other conventional supported Ni catalysts were also prepared. The catalytic performance and durability in the steam reforming of ethanol were examined and compared for various Ni catalysts, including the smectite-derived and conventional supported Ni catalysts.

Smectite is one of the layered clay minerals, and it can be synthesized by a simple hydrothermal method [16]. There is a layer consisting of one octahedral sheet sandwiched by two tetrahedral sheets, and it is possible to introduce divalent or trivalent cations, such as Mg^2+^, Zn^2+^, Al^3+^ and Fe^3+^ in a framework of an octahedral sheet by using different precursors. The synthetic smectite materials can serve as catalysts and supports due to their high surface area and their flexible structural features, as demonstrated by a number of studies, including hydrogenation [17,18], hydrogenolysis [18], synthesis of dimethyl carbonate [19], transesterification and Knoevenagel reactions [20], steam reforming of acetic acid [21], dry reforming of methane with carbon dioxide [22], selective methanation of carbon monoxide [23] and others [24,25]. The metal species in the smectite structure may be reduced, move to the surface layer and form metal clusters/crystallites on the wall of pores through heat treatment with H_2_, which can thus serve as dispersed metal catalysts. These morphological features of metal-containing smectite-derived materials may give some beneficial effects to the synthesis of Ni catalysts for the steam reforming of ethanol, as well. Therefore, the present work has been undertaken to improve the activity and durability of Ni-based catalysts by using Ni-containing smectite-derived materials as catalyst precursors.

## 2. Results and Discussion

The Mg- and Ni-containing smectite samples are abbreviated as SM and SM(Ni) (or SM(Ni35) to indicate the Ni content), respectively, in this work. Table 1 shows the textual properties of Ni catalysts prepared (after reduction at 600 °C), including SM(Ni35), a smectite-derived material containing Ni in 35 wt % (Entry 1), and other supported Ni catalysts prepared by impregnation using different supports and/or different Ni loadings (Entries 2–7). Among the catalysts prepared, SM(Ni35) gave the highest surface area of 493 m^2^·g^−1^, and its Ni crystallite size of 5.4 nm was the smallest among the samples with the same Ni loading (Entries 1, 4–7). However, its CO uptake was 30.5 µmol·g^−1^, which was smaller than 41.0 µmol·g^−1^ for Ni35/SM, meaning that the amount of exposed surface Ni sites in the SM(Ni35) was smaller in spite of its smaller Ni crystallite size (Entries 1, 4). These results indicate that some Ni species in SM(Ni35) remain in the framework of the smectite structure even after reduction. Generally, the crystallite size of supported metal tends to increase with the metal loading, as shown in the case of Ni/SM (Entries 2–4), and so, it is difficult to synthesize metal nanoparticles in a high metal loading. However, in the present case of Ni-containing smectite-derived material, the crystallite size of Ni was smaller than those of the Ni catalysts impregnated in smaller loadings, Ni05/SM and Ni10/SM (Entries 1–3). Hence, the Ni nanoparticle was able to be produced in a high metal loading when a smectite-derived material was used as a starting precursor.

**Table 1:** Textual properties of the different Ni catalysts prepared.

Entry	Catalyst	Ni Loading (wt %)	Surface Area (m^2^·g^−1^)	CO Uptake ^a^ (μmoL·g^−1^)	d_Ni_^b^ (nm)
1	SM(Ni35)	35	493	30.5	5.4
2	Ni05/SM	5	478	2.4	6.6
3	Ni10/SM	10	457	7.3	7.5
4	Ni35/SM	35	293	41.0	21.0
5	Ni35/MgO	35	25	23.8	19.0
6	Ni35/SiO_2_	35	323	20.2	37.0
7	Ni35/Al_2_O_3_	35	100	35.9	22.0

^a^ The amount of CO adsorbed on the catalyst measured by CO chemisorption; ^b^ crystallite size of the Ni particle calculated from XRD results.

**Figure 1:**
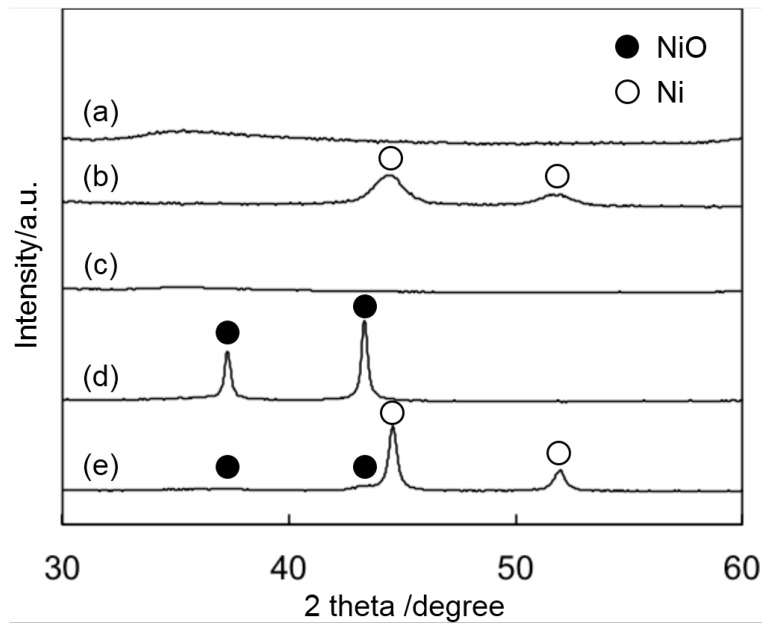
XRD patterns of SM(Ni35) before reduction (a) and after reduction (b); SM after calcination (c); Ni35/SM before reduction (d) and after reduction (e).

The formation of Ni nanoparticles in SM(Ni35) was examined by XRD (Figure 1). Two peaks assigned to the Ni metal were seen for SM(Ni35) after reduction, whereas no peak was observed before reduction (Figure 1a,b), indicating that Ni species were contained in a framework of SM(Ni35) just after synthesis, and these moved to the surface during the reduction process, forming nanoparticles. On the other hand, sharp peaks assigned to NiO were observed for Ni35/SM before reduction (Figure 1d) and changed almost to Ni metal after reduction (Figure 1e) with no change in the Ni crystallite size. Therefore, there are different mechanisms for the formation of Ni nanoparticle on SM(Ni35) and in Ni35/SM. For SM(Ni35), Ni species in a framework moved to the surface during reduction, but a strong interaction between Ni and the framework inhibited the significant sintering of Ni species, resulting in the formation of small Ni nanoparticles. For Ni35/SM, the crystallite size of Ni was determined before the reduction process, which was likely to depend on the calcination conditions.

### 2.1. Catalytic Activity of Smectite-Derived and Conventional Supported Ni Catalysts

**Figure 2:**
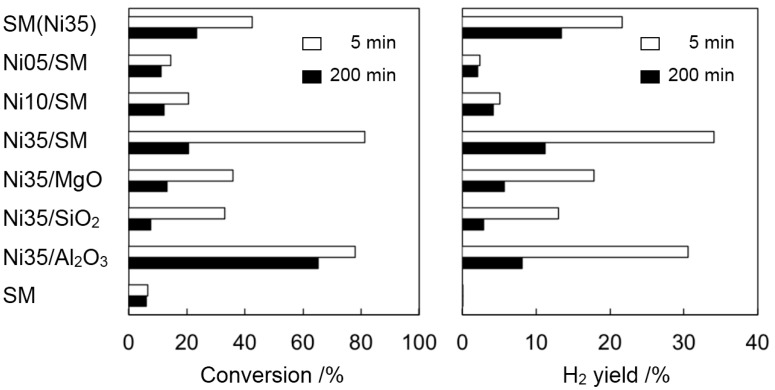
Total conversion and H_2_ yield for steam reforming of ethanol over different Ni catalysts at 500 °C after 5 min (□) and 200 min (■).

**Figure 3:**
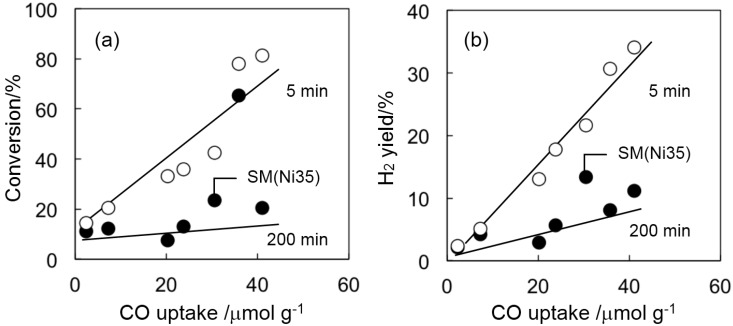
Relationship between the amount of surface Ni (CO uptake measured by CO chemisorption) and either total conversion (**a**) or H_2_ yield (**b**) after the reaction time of 5 min (○) and 200 min (●).

Steam reforming of ethanol was carried out at 500 °C over the Ni catalysts prepared, which were compared to the total conversion and H_2_ yield at the initial stage (after 5 min) and at the later stage (after 200 min) (Figure 2). For a comparison, the catalytic performance of SM support was also measured. At the initial stage, a supported Ni catalyst of Ni35/SM gave the highest catalytic performance in both ethanol conversion and H_2_ yield among the catalysts used, but a remarkable catalyst deactivation was observed after 200 min. For the smectite-derived SM(Ni35) catalyst, however, initial conversion and H_2_ yield were lower than those of Ni35/SM, but, at the later stage of the reaction, higher catalytic performance was obtained despite the lower amount of surface Ni sites determined by CO chemisorption (Entries 1, 4 in Table 1). These results indicate that the durability of the Ni catalyst was improved by using a Ni-containing smectite as a precursor. After 200 min of reaction, the highest H_2_ yield was achieved over SM(Ni35) catalyst. It should be mentioned that an Al_2_O_3_-surpported catalyst of Ni35/Al_2_O_3_ gave a high conversion even after a long-time reaction, but the main carbon-containing product changed from CO_2_ to ethylene, which should be produced by the dehydration of ethanol on the acid sites of Al_2_O_3_ support, but not on Ni particles. Namely, undesired side reactions occurred, and so, Ni35/Al_2_O_3_ was losing its activity for the desired H_2_ production by the steam reforming of ethanol, as shown in Figure 2. The total ethanol conversion and H_2_ yield were plotted against the amount of exposed Ni sites measured by CO chemisorption in Figure 3. A clear correlation between the amount of surface Ni and either conversion or H_2_ yield was observed at the initial stage, meaning that the initial reaction rate simply depended on the amount of active sites, and these catalysts lost their activity after 200 min of reaction to a similar extent (Figure 3a). However, the extent of catalyst deactivation was relatively lower in the case of using SM(Ni35) compared with the other catalysts prepared by impregnation. That is, the durability of the catalyst was improved by using the Ni-containing smectite as the precursors.

### 2.2. Catalyst Deactivation

**Figure 4:**
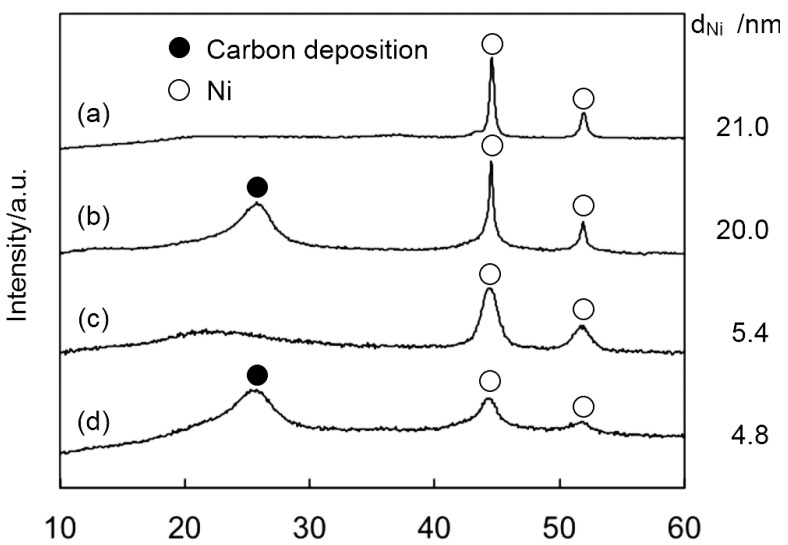
XRD patterns and the crystallite sizes of Ni35/SM before (a) and after reaction (b); and SM(Ni35) before (c) and after reaction (d) at 500 °C for 6 h.

**Figure 5:**
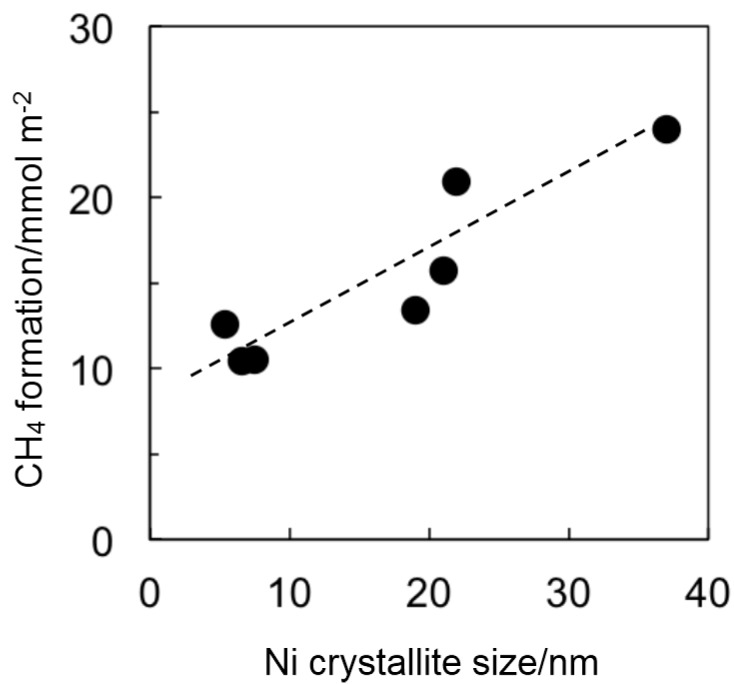
Relationship between the crystallite size of Ni particles and the amount of CH_4_ formed (the amount of carbon deposits) per exposed Ni surface area.

XRD results for Ni35/SM and SM(Ni35) catalysts after reaction showed the peak at 26°, which was assignable to carbon deposition (Figure 4). On the other hand, the crystallite size of Ni particles determined by XRD did not change, as shown in Figure 4, indicating that the sintering of Ni was unlikely to occur under the present reaction conditions. Therefore, we conclude that the carbon deposition was the main cause of the catalyst deactivation. Carbon deposition was observed for all Ni catalysts used, and the amounts of the carbon deposited were determined from the total amount of CH_4_ detected during H_2_ heat treatment. Interestingly, the amount of carbon deposition per Ni surface area tended to decrease with the decreasing crystallite size of Ni particles (Figure 5), meaning that the formation of the Ni nanoparticle not only results in the increase of exposed Ni surface area, but also contributes to the decrease of the carbon deposition.

**Figure 6:**
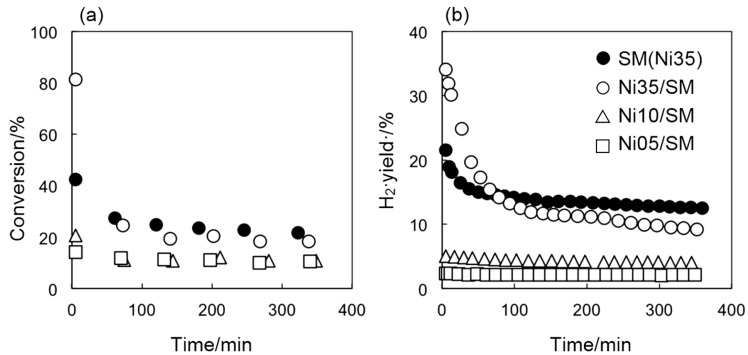
Time profiles of total conversion (**a**) and H_2_ yield (**b**) over different Ni catalysts at 500 °C.

Figure 6 gives the time profiles of the total conversion and H_2_ yield over SM(Ni35) and other Ni-loaded catalysts prepared by impregnation. For the Ni35/SM catalyst, remarkable catalyst deactivation was observed within a first 100 min, and the deactivation continued to occur gradually after 100 min. Such catalyst deactivation was not observed for Ni05/SM and Ni10/SM, which have small Ni nanoparticles, as shown in Table 1, implying that a rapid catalyst deactivation due to carbon deposition is unlikely to occur on a small Ni nanoparticle. For SM(Ni35), the formation of Ni nanoparticle in SM(Ni35) may be responsible for its relatively stable catalytic performance for both conversion and H_2_ yield during the reaction.

**Figure 7:**
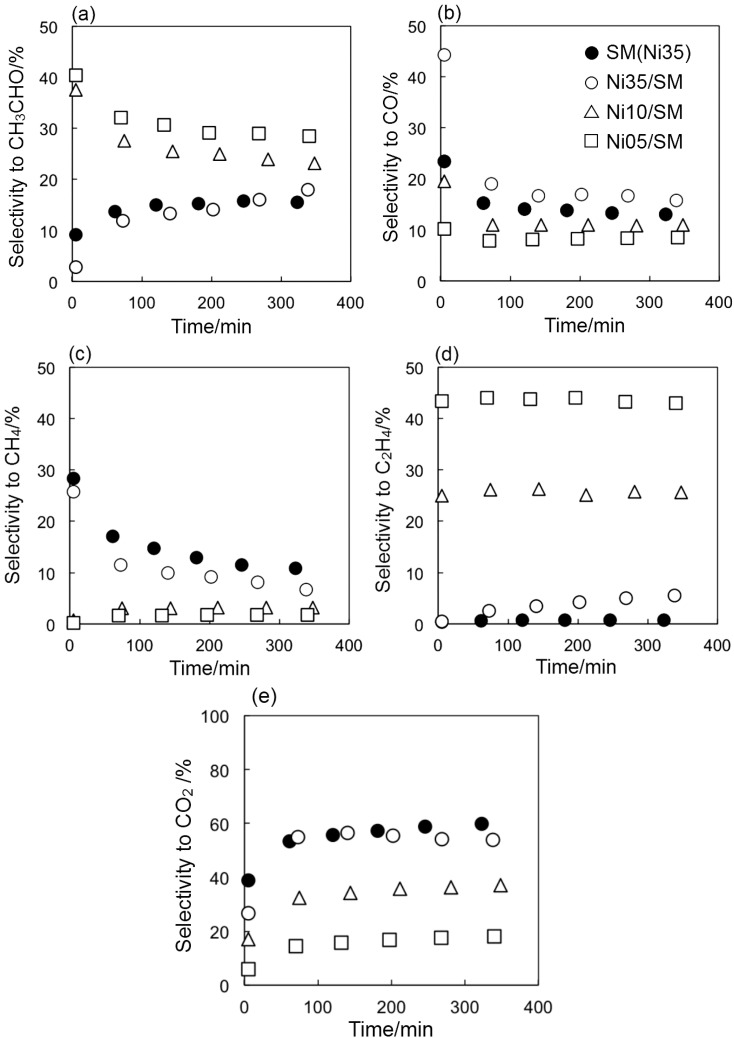
Time profiles of selectivity to CH_3_CHO (**a**); CO (**b**); CH_4_ (**c**); C_2_H_4_ (**d**) and CO_2_ (**e**) over different Ni catalysts given at 500 °C.

Time profiles of the product selectivity were examined to further discuss the carbon deposition (Figure 7). At the beginning of the reaction over SM(Ni35) and Ni35/SM, high selectivity to CO and CH_4_ (Figure 7b,c) and low selectivity to CH_3_CHO and CO_2_ (Figure 7a,e) were obtained; the dehydrogenation of ethanol to C_1_ species (Equation (2)) and the decomposition of CH_3_CHO (Equation (3)) took place rapidly, but further reaction of C_1_ species with water, the steam reforming of CH_4_ (Equation (4)) and the water gas shift reaction (Equation (5)) should be slower than the former reactions. During the reaction, the selectivity to CH_3_CHO and CO_2_ tended to increase (Figure 7a,e) while that to CO and CH_4_ decreases (Figure 7b,c), implying that the decomposition of CH_3_CHO to CO and CH_4_ (Equation (3)) was suppressed by carbon deposition compared with the other reactions. Therefore, one can say that CO and CH_4_ formed from CH_3_CHO induced the side reactions to form the carbon deposition on the catalyst (Equations (8) and (9)), which suppressed the further decomposition of CH_3_CHO and resulted in the catalyst deactivation. Vicente *et al.* [26,27] investigated the steam reforming of ethanol over Ni/SiO_2_ catalyst, and they noted that CO and CH_4_ formed by the decomposition of CH_3_CHO were the precursors of the filamentous coke deposited on the Ni surface. For the three catalysts prepared by impregnation, the selectivity to CO against that to CH_4_ was higher, and this means that the rate of CH_4_ steam reforming is faster than the water gas shift reaction. For the smectite-derived SM(Ni35) catalyst, however, the selectivity to CO against that to CH_4_ was relatively lower compared with the case of Ni35/SM, which indicates that the reactivity of CO was enhanced by Ni nanoparticles in the framework of the smectite structure.

It is known that the decomposition of C_2_H_4_ also causes carbon deposition (Equation (7)). The formation of C_2_H_4_ was observed for the catalysts prepared by impregnation, but no or little catalyst deactivation was observed over low Ni-loaded catalysts (Figure 7d). To reveal the active site for the formation of C_2_H_4_, the SM sample, including Mg instead of Ni, was used for the steam reforming of ethanol. A C_2_H_4_ selectivity of 96% was observed at a conversion of 6.5%. Therefore, we conclude that the dehydration of ethanol is likely to occur on the SM support, and this can also explain the increase of the selectivity to C_2_H_4_ as the Ni loading is reduced. Nishiyama and Shirai [28,29] measured FTIR spectra of pyridine adsorbed on smectites and indicated the presence of weak Lewis acid sites and the absence of Brønsted ones, and hence, these acidic properties of the smectite surface induced the dehydration of ethanol, but not for the decomposition of C_2_H_4_, for which the strong acid sites are needed [30,31,32]. It is noteworthy that the formation of C_2_H_4_ was not observed during the reaction over SM(Ni35), whereas the selectivity to C_2_H_4_ gradually increased over Ni35/SM (Figure 7d). These results indicate that the Ni-containing smectite structure is useful for the suppression of the side reaction of ethanol to C_2_H_4_ and contributes to the increase of the selectivity to the steam reforming reaction. These features of Ni-containing smectite materials would be beneficial for other catalytic reactions, in which the carbon deposition and/or the dehydration should be avoidable.

## 3. Experimental Section

### 3.1. Catalyst Preparation

Smectite materials were prepared in the same manners as described elsewhere [17,18]. Aqueous solutions of water glass (Nippon Chemical, Tokyo, Japan) and NaOH (Wako Pure Chemical, Osaka, Japan) were mixed, and an aqueous solution of MgCl_2_ (Wako) was dropped into the mixed solution until the molar ratio of Si/Mg became 8/6 while stirring. After stirring for 12 h under ambient conditions, the resultant solid material was separated by filtration and washed with distilled water. The solid material was put into a certain volume of water (20 cm^3^), of which the pH was 7.34, and the slurry obtained was put into a stainless steel autoclave (100 cm^3^). After a heat treatment at 150 °C for 2 h, the solid material produced was separated by filtration and washed with distilled water, dried at 80 °C overnight and calcined at 300 °C for 1 h. Nickel-containing smectite material was also prepared with similar procedures using NiCl_2_ (Wako) instead of MgCl_2_, in which the amount of Ni included was 35 wt %. The SM(Ni) sample was reduced in a stream of 4% H_2_ in N_2_ at a rate of 50 cm^3^·min^−1^ and at increasing temperatures at a rate of 10 K·min^−1^ up to 700 °C, at which it was further reduced for 1 h. SM-supported Ni catalyst was prepared by the impregnation method. The material was impregnated with an aqueous solution of Ni(NO_3_)_2_·6H_2_O (Wako) at 70 °C under reduced pressure, dried at 110 °C overnight and calcined at 500 °C for 3 h. The loadings of Ni prepared were 5, 10 and 35 wt %. SiO_2_ (GL Science, Tokyo, Japan), Al_2_O_3_ (Catalysis Society of Japan, JRC-ALO4) and MgO (Kishida Chemical, Osaka, Japan) were also used for supports, and Ni was loaded onto these supports in 35 wt % by impregnation using NiCl_2_.

### 3.2. Catalyst Characterization

Total surface area was measured by nitrogen adsorption at −196 °C on Quantachrome NOVA 1200 (Version 7.01). The samples were pretreated by evacuation at 150 °C for 2 h. The structural properties of Ni species before and after reduction were examined by X-ray diffraction (XRD) on a JEOL JDX-8020 with Ni-filtered CuKα radiation. The crystallite size of Ni was determined by Scherrer’s equation. The area of exposed Ni species was measured by CO pulse chemisorption (BEL Japan BEL-METAL, Osaka, Japan) at 50 °C using a pulse of 5% CO in He. Prior to the chemisorption, the samples were treated in a stream of 5% H_2_ in He at 600 °C for 1 h.

### 3.3. Steam Reforming of Ethanol

The steam reforming of ethanol was carried out in a glass fixed-bed flow reactor at atmospheric pressure. A catalyst sample (0.01 g) was placed in the reactor, reduced in a stream of 4% H_2_ in N_2_ at a rate of 50 cm^3^·min^−1^ and at increasing temperatures up to 600 °C and further reduced at this temperature for 1 h. After cooling to room temperature in N_2_, the reactor was heated to a reaction temperature of 500 °C. A mixture of ethanol and water diluted with N_2_, of which the partial pressures were 0.05 and 0.15 atm, respectively, was introduced at a total flow rate of 200 cm^3^·min^−1^. All of the products after the reaction were analyzed by gas chromatographs with thermal conductivity detector (TCD) and flame ionization detector (FID). The total conversion of ethanol was determined from the concentration measured before and after reaction. The selectivity to the carbon-containing products was determined on the carbon basis, and the selectivity to CO_2_ was used as a measure of the selectivity to the desired steam reforming of ethanol. The H_2_ yield was determined from the amount of H_2_ formed divided by the stoichiometric maximum amount of H_2_ in the steam reforming of ethanol (Equation (1)) to be obtained under the conditions used.

### 3.4. Estimation of Carbon Deposition

The amount of carbon deposited on the surface of Ni catalysts was determined as follows. A used catalyst sample was subjected to the thermal treatment in a stream of 4% H_2_ in N_2_ at a rate of 100 cm^3^·min^−1^ and at increasing temperatures up to 700 °C and at this temperature for a few hours. The total amount of CH_4_ evolved during the treatment was measured.

## 4. Conclusions

The stability of Ni catalyst for the steam reforming of ethanol was improved by the use of a Ni-containing smectite-derived material as a precursor. The decomposition of CO and CH_4_ to the carbon deposition on Ni particles causes significant catalyst deactivation, but it can be suppressed to an even larger extent for the Ni-containing smectite-derived catalyst. Furthermore, the side reaction of the dehydration of ethanol to C_2_H_4_, which occurs on the acid sites of the support, was completely inhibited. The smectite-derived catalyst contains smaller and more stable Ni crystallites compared to other conventional supported Ni catalysts, which should be related to the formation of exposed Ni crystallites from the smectite bulk structure, different from that via impregnation, in which Ni is loaded onto support materials from outside.
